# Visual Analytics of Gaze Data with Standard Multimedia Players

**DOI:** 10.16910/jemr.10.5.4

**Published:** 2017-11-20

**Authors:** Julius Schöning, Christopher Gundler, Gunther Heidemann, Peter König, Ulf Krumnack

**Affiliations:** Institute of Cognitive Science, Osnabrück University, Germany

**Keywords:** eye-tracking, gaze data, visualization, visual analytics, multimedia container

## Abstract

With the increasing number of studies, where participants' eye movements are tracked while watching videos, the volume of gaze data records is growing tremendously. Unfortunately, in most cases, such data are collected in separate files in custom-made or proprietary data formats. These data are difficult to access even for experts and effectively inaccessible for non-experts. Normally expensive or custom-made software is necessary for their analysis. We address this problem by using existing multimedia container formats for distributing and archiving eye-tracking and gaze data bundled with the stimuli data. We define an exchange format that can be interpreted by standard multimedia players and can be streamed via the Internet. We convert several gaze data sets into our format, demonstrating the feasibility of our approach and allowing to visualize these data with standard multimedia players. We also introduce two VLC player add-ons, allowing for further visual analytics. We discuss the benefit of gaze data in a multimedia container and explain possible visual analytics approaches based on our implementations, converted datasets, and first user interviews.

## Introduction

It is still common practice to store gaze information belonging to video stimuli next to the video file in custom file formats. In addition to proprietary formats, where the data structure of the gaze information is unknown, the data structures are strongly customized—sometimes unique—and stored in a diversity of formats, e.g., plain text, XML, Matlab MAT format, or even binary. As a consequence, special tools for accessing, visualizing and analyzing these data and stimuli are needed. For the general audience, specialized software tools, which are expensive or require compilation, are a major obstacle and often prohibit accessing the data.

An open and standardized exchange format will overcome this problem and will form the basis for visualization and visual analytics (VA) software. Thus, why not encapsulate gaze data alongside the stimuli material in a joint container? For storing text plus metadata, this has become common practice—a well-known example is the PDF container. For the encapsulation of multimedia content comprising video, audio and metadata, such as subtitles, audio comments, and interactive features, multimedia container formats were specified. Examples are the open container formats ( 30 ) (OGG), MPEG-4 ( 10 ), or the Matroška container ( 12 ) (MKV). Housing multimedia content in a single file, which can be easily archived, has fixed and validated synchronization of different data streams, can be played by standard multimedia players, and can be streamed via the Internet. If gaze data or other participant-related data, such as EEG traces, are encapsulated in such container formats, the accessibility will be improved for both experts and the general audience, in scientific publications, or in popular science video demonstrations^1^.

We present concepts and first software tools for creating, analyzing, and decomposing multimedia containers of eye-tracking research data. Our aim is that video eyetracking data can be stored in common multimedia containers, carrying (i) the video stimuli, (ii) the gaze trajectories of multiple participants, and (iii) other videorelated data. With a strong focus on gaze data, we evaluate current multimedia containers that support a variety of video, audio, and subtitle data formats. We want to find out if such containers can provide instantaneous visualization and get VA support in standard media players. By contributing our code extensions to official development repositories, our long-term aim is to establish a standard format. Based on that format, research data could be encoded uniquely and will benefit various fields, ranging from training neuronal networks based on human sensory data over highlighting objects in movies for visually impaired people to creating auditory displays for blind people. Further, VA on gaze data may experience a boost once a standard format exists that allows sharing data and results, and combining gaze data with other metadata.

Building on previous work on merging video and eyetracking data ( 19 ), we focus on the following aspects in the present paper: i) a more detailed discussion of feasible data formats for metadata description, timed data representation, and multimedia encapsulation (cf. Section Data Formats and Suitable Data Formats for Gaze Data), ii) the description and implementation of software tools for creating and decomposing multimedia containers with gaze data as well as our first two add-ons for VA on gaze data using the VLC media player (cf. Section Software Tools for Gaze Data in Multimedia Containers), and iii) a discussion about benefits of our approaches highlighted in user interviews, and a list of available data sets (cf. Section User Study). Based on a comparison of available container formats suitable for housing sensory and other relevant data, we conclude by discussing possible future work in the field of VA and computer vision, where gaze data in multimedia containers have a strong impact.

## Data Formats

By listing available video eye-tracking data sets ( 27 , cf. Section 2.2), the diversity of formats, they are stored in, e.g., plain text, XML, Matlab MAT format, or even binary, is quite impressive. Any of these formats provides instantaneous visualizations, and the stimulus sequence can be streamed together with metadata, like gaze trajectories and object annotations. In contrast, the domains of DVD, Blu-ray, or video compression can provide formats with efficient mechanisms for visualization and streamability via the Internet. Therefore, it is reasonable to evaluate the formats used in these domains and analyze whether one of these fits the requirements for storing research data. These requirements include e.g. storing the stimuli, as well as all relevant metadata, providing opportunities for visualization, and, if possible, VA within a standard multimedia player.

### Metadata Formats

Computer vision algorithms still have great difficulties gaining semantic content from video sequences. Hence, VA of gaze trajectories of subjects performing, e.g., a visual search task, may provide insights for designing better algorithms. Gaze trajectories might also serve as training data for deep learning approaches if the data representations of several data sets are compatible. However, combining, extending, exchanging, and analyzing gaze data from different repositories is tedious, timeconsuming, as well as inefficient, due to the lack of a standardized format.

With the rise of the semantic web, standards for metadata were introduced, which have become quite popular. The most general standard for expressing metadata is the resource description framework (RDF) ( 25 ). Its well-defined formal syntax and distributed representation allow statements about resources, i.e., virtually everything that can be uniquely described. The sparse predefined vocabulary of RDF requires extension if RDF is used in a new domain. By now, several vocabulary schemes for various domains exist, but there is, to our knowledge, no scheme for video content. Further, the temporal and spatial structure of video sequences differs from the majority of vocabularies. Therefore, a new vocabulary scheme has to be defined for fitting eye-tracking data set.

For annotating time-continuous data with different types of metadata, the Continuous Media Markup Language (CMML) ( 16 ) was developed. Similar to HTML description, it provides markup facilities for timed objects, which are ideal for the integration of multimedia content on the Internet. In a predefined vocabulary, CMML provides textual descriptions, hyperlinks, images (e.g., keyframes), and—in the form of value pairs—other unspecified metadata. By using this vocabulary, temporal metadata of video and audio sequences can be represented. Unfortunately, the spatial structure, i.e., reference to pixels, regions, and areas is not provided by CMML.

Probably the most advanced metadata framework for multimedia content is MPEG-7, which has been developed by the Moving Picture Experts Group. This Multimedia content description interface defined in the ISO/IEC 14496-3 standard, specifies a set of mechanisms for describing as many types of multimedia information as possible. However, this specification has been criticized and has lost support because of its lack of formal semantics. This causes ambiguity, leading to interoperability problems, and hinders a widespread implementation and a standard interpreter ( 14, 2 ).

Nevertheless, MPEG-7 can describe multimedia content at different degrees of abstraction and is designed as an extensible format. Using its own “Description Definition Language” (DDL – an extended form of XML Schema), its defines connected vocabularies on all levels of abstraction. The basic structure of the vocabulary is focused on GRID LAYOUT}, TIME SERIES, MULTIPLE VIEW, SPATIAL 2D COORDINATES, and TEMPORAL INTERPOLATION. For object labeling, as well as continuous gaze trajectories, the feature TEMPORAL INTERPOLATION could be useful. Using connected polynomials, it allows temporal interpolation of metadata over time and forms the basis of the SPATIO-TEMPORAL LOCATOR which can be used for describing, e.g., the bounding box of an object in the complete video sequence.

### Timed Text

Like a video transcript, timed text ( 26 ) assigns text labels to certain time intervals based on time-stamps and is typically used for subtitling of movies, as well as captioning for hearing impaired or people lacking audio devices. The simplest grammar of timed text consists of a time interval (start and end time) and the text to be displayed. The popular SubRip format (SRT) sticks to that grammar only. Enhancing this temporal grammar with spatial features, the second edition of the Timed Text Markup Language 1 (TTML1) ( 24 ) introduces a vocabulary for regions. Nowadays, the amount of variation within timed text formats makes them hardly interoperable. Therefore, a large set of decoders and rendering frameworks have emerged for supporting the compatibility of media players.

### Subtitle and Caption Formats

With the objective of providing a modern format for encoding subtitles, the open universal subtitle format (USF) was specified ( 15 ). For being comprehensive, it tries to form the superset of features from different existing subtitle formats. However, in contrast to existing subtitle formats, it provides a XML-based representation which is flexible, human readable, portable, Unicode compatible, and hierarchical structured and further it provides an easy management of entries. Designed for being rendered by the multimedia players, it is possible to adapt the display style (color, size, position, etc.) to fit the needs of the viewer. In contrast to pre-rendered subtitles, like the VobSub format, this gives the opportunity for adjusting the visualization of the metadata according to the analysis task. However, not every multimedia player supports the rendering of subtitles during playback.

Regrettably, the open source USF project has become private and its community has lost all influence on the development. In addition, the documentation of the latest version 1.1 has been temporary removed from the Internet repository. In this working version 1.1, some parts, including the draw commands, were marked as under development, but in addition to visualization of metadata on top of the video sequence, USF provides comment tags to allow storing additional information that cannot be visualized.

Together with a subtitle editor, the Sub Station Alpha (SSA) has been introduced and is being widely used by fansubbers. Because of its widespread use, the support of SSA and its extended version V4.00+ ( 22 ), known as Advanced SSA (ASS), is integrated in almost every multimedia player. In addition to SSA, ASS includes simple drawing commands like straight lines, 3^rd^ degree Bezier curves, and 3^rd^ degree uniform b-splines, which are probably sufficient to visualize gaze points, objects annotation, etc. on a certain frame. The latest version of today’s video players support the ASS drawing commands.

### Multimedia Container

**Figure 1:**
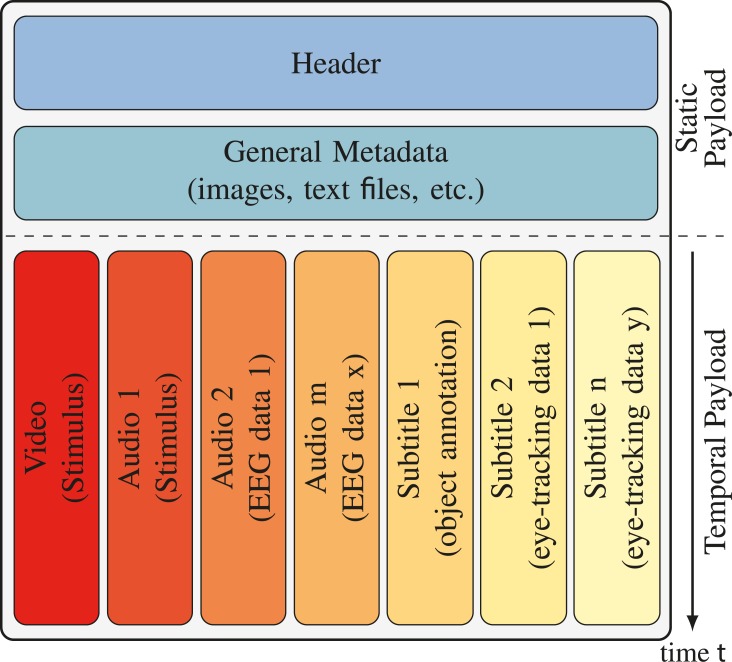
In general, the structure of a multimedia container is divided into two parts based on the temporal dependencies of the content. Content without temporal structure, like the header, general metadata, as well as attachments are stored before temporal related content such as videos, audios, subtitles, and other metadata. As illustrated, the subtitle tracks can be used for the visualization of gaze data, object annotations, etc. and the audio tracks for sonification of, e.g., EEG data. For watching these multimedia containers via the Internet, only the non-temporal content has to be transmitted before playing, while the temporal content is transmitted during playing.

Multimedia containers are capable of housing any multimedia content in a single container, which then can be used for storing, distributing, broadcasting, and archiving. Usually, multimedia containers have multiple parallel tracks such as video, audio, and subtitle tracks encoded by their format standards. Unlike classical data archives such as ZIP or TAR, multimedia containers take the temporal nature of the tracks—their payload—into account. As illustrated in Figure 1, the temporal payload is synchronized to support streaming and playback without decomposition of the container. To implement these features, the muxer, which creates the multimedia container from the data, must be able to understand, as well as encode, the data into the temporary structure of the container. Thus, there is a strong dependency between video, audio, subtitle, etc. data formats and container formats. In summary, not every payload is suited for every container format.

Common multimedia containers used for DVDs are the VOB and EVO formats, which are based on the MPEGPS standard. Specified in MPEG-4 (ISO/IEC 14496, Part 14) ( 10 ), the more modern MP4 container format was specified to hold video, audio, and timed text data. In accordance with the other standards of MPEG, MP4 encapsulates only video, audio, and subtitle formats specified by the MPEG.

Originally put forward by the non-profit Xiph.Org ( 30 ) foundation, the free OGG container format is designed for streaming Vorbis encoded audio files and is nowadays supported by many portable devices. Through the launch of Theora and Dirac video formats, OGG has become a more and more popular format for streaming web multimedia content.

With the aim to allow an easy inclusion of different types of payloads, the open Matroška container format (MKV) ( 12 ) is a flexible and widely used container. Also, it serves as a basis for the WEBM format, which is being pushed forward to establish a standard for multimedia content on the web.

The inclusion of timed metadata that goes beyond subtitle formats into multimedia containers seems to be discussed only sporadically. Embedding CMML descriptions in an OGG container is the only approach discussed ( 29 ). However, the lack of CMML for supporting spatial features like region markup and object boundaries will rule out its use in present standard multimedia players.

For metadata and stimuli, probably the most matured specified approach is the embedding of MPEG-7 ( 9 ) data into MP4 ( 10 ) containers. Nonetheless, there are to our knowledge still no multimedia players with MPEG-7 support.

## Suitable Data Formats for Gaze Data

What kind of data format would be best suited for gaze trajectories of several subjects, the video stimulus itself, and optional metadata like object annotations? In the previous section, we gave a brief overview of the diversity of formats of metadata and multimedia containers. Fostered by the advance of the semantic web and other digital technologies, the diversity keeps growing. While general formats, like RDF ( 25 ), are well established, they are not geared towards the description of temporal content like video sequences, and hence miss the required vocabulary. To avoid the introduction of new formats, we suggest three approaches to a standard format for gaze data on video stimuli. The first approach is the extension of a general metadata formalism like RDF with video, audio, and eye-tracking, as well gaze data vocabulary. As the second approach, we use a welldefined standard for metadata like MPEG-7 along with the implementation of proper multimedia player extensions. Third, we present an ad hoc approach that utilizes existing technologies, e.g., for subtitles or captions, to store gaze data and object annotations.

The first approach, i.e., the development of a specialized eye and gaze tracking format on the basis of a well-established metadata framework like RDF yields the obvious advantage that it can build on the vast amount of software libraries and tools that support storage, management, exchange, and a certain type of reasoning over such data. The main drawback is that these formats lack any form of support for the video, as well as the audio domain, and do not even provide basic spatial or temporal concepts. The development of a specialized vocabulary to describe gaze tracking scenarios would mean a huge effort. Additionally, it would have to include the implementation of tools for visualization. Desirable features, like streamability, do not seem to fit the original static nature of these data formats well. Furthermore, given the specificity of the field of eyetracking, a wide support by common multimedia players seems unlikely.

Using a specialized, detailed standard for video metadata like MPEG-7 is the second possible approach. MPEG-7 provides a well-defined and established vocabulary for various annotations, but lacks a vocabulary for gaze data. Nevertheless, MPEG-7 supports the description of points or regions of interest in space and time, and hence, seems well suited to store the essential information of eye-tracking data. Unfortunately, no standard media player (like VLC, MediaPlayer, and Windows Media Player) currently seems to support the visualization of MPEG-7 video annotations. Hence, one would have to extend these players to visualize embedded eyetracking data—fortunately, one can build on existing MPEG-7 libraries ( 3 ). We think that, when implementing such multimedia player extensions, one should aim at a generic solution that can also be used to visualize other MPEG-7 annotations, as this would foster development, distribution, and support.

Even though the first two approaches seem better suited in the long run, they appear neither realizable with manageable effort nor on a short time scale. Hence, in the remainder of this paper, we focus on the third approach, which allows for the quick development of a prototype to demonstrate the idea, concepts, possibilities, and to gain experience in its application. The idea is to adopt existing technologies, like subtitles, captions, audio tracks, and online links ( 4 ), already supported by existing media players. Although these technologies are not yet geared towards the storage and presentation of gaze data, all important features can be realized based on these formats. Reusing or “hijacking” formats has the benefit that they will be widely supported by current multimedia players. Even though there appears to be no general drawing framework, some subtitle formats include drawing commands that allow highlighting regions in a scene. These commands can also be used for visualizing gaze data or for auditory display of EEG data. Using a player’s standard methods for displaying subtitles and switching between different languages, one can then display gaze data and switch between data of different subjects. Using player add-ons, simple VA tasks are possible by reusing tracks of the multimedia container.

When putting metadata into a reused format, one must be careful that no information is lost. Additionally, one should bear in mind that the original format was designed for a different purpose, so it may not have out-of-the-box support for desirable features, e.g., simultaneous display of gaze points of multiple subjects. We chose to use USF for three main reasons: First, its specification considers possible methods for drawing shapes, a prerequisite for instantaneous visualization with a normal multimedia player. Second, it allows for storing additional data, a necessity for carrying all eye-tracking data so that expert visualization with specialized tools is possible from the same single file. Finally, and most important, USF is— like the preferable MPEG-7—a XML-based format and thereby capable of holding complex data structures. However, although basic USF is supported by some existing media players, the drawing commands are not implemented. We provide an extension for the VLC media player that implements some drawing commands. Also, we provide a converter to the ASS format, which is widely supported, including drawing commands, due to the free ASS library, thereby allowing out-of-the-box visualization of eye-tracking data that works with many current multimedia players. However, its plain text format is too restricted to hold all desirable information. Both approaches will be discussed in more detail in the next section.

## Software Tools for Gaze Data in Multimedia Containers

Beyond two prototypes of multimedia containers where we embedded gaze data and object annotation using two different subtitle formats, we present in this section our new tool for converting metadata along with the stimulus video sequence into a MKV container. Followed by a brief introduction of a tool for decomposing a multimedia container, we present add-ons to VLC which allow simple VA on multimodal data—here gaze data— using a standard media player.

### Container Prototypes

For the encapsulation of metadata, we implement two kinds of prototypes where the subtitle track is reused as a carrier of metadata. Our first prototype based on USF encapsulates the complete gaze metadata without loss and can be visualized in a modified version of the VLC media player. Based on the ASS format, the second one is only enabled to carry selected metadata for visualization, but these visualizations can be displayed by current media players, as well as standalone DVD players.

**Metadata as USF.** In order to use USF for encapsulating eye-tracking data, we analyzed which features of USF are available in the latest releases of common multimedia players. One of the most common media players is the VLC media player. The current developer version 3.0.0 already supports a variety of USF attributes, which are text, image, karaoke, and comment. The latest USF specification introduces an additional attribute shape that is still marked as under development, although this specification is already quite old. Since gaze data is commonly visualized with simple geometric shapes, like circles and rectangles, the use of the shape attributes for instantaneous gaze visualization of subjects seems to be quite appropriate.

Since the exact specification of the shape attribute is, as mentioned above, not complete, we particularized it on rectangles, polygons, and points, as illustrated in Listing 1. These simple geometric shapes were taken as first components in order to visualize a multitude of different types of elements. Point-like visualizations are useful to describe locations without area information, e.g., for gaze point analysis in eye-tracking studies. Rectangles are most commonly used as a bounding box for object of interest annotations, whereas polygons provide a more specific, but complex way of describing the contour of an object. Further, as can be seen in Figure 9, one can use different geometric shapes to differentiate between, e.g., saccades marked by circles and fixations marked by rectangles.

**Listing 1:**
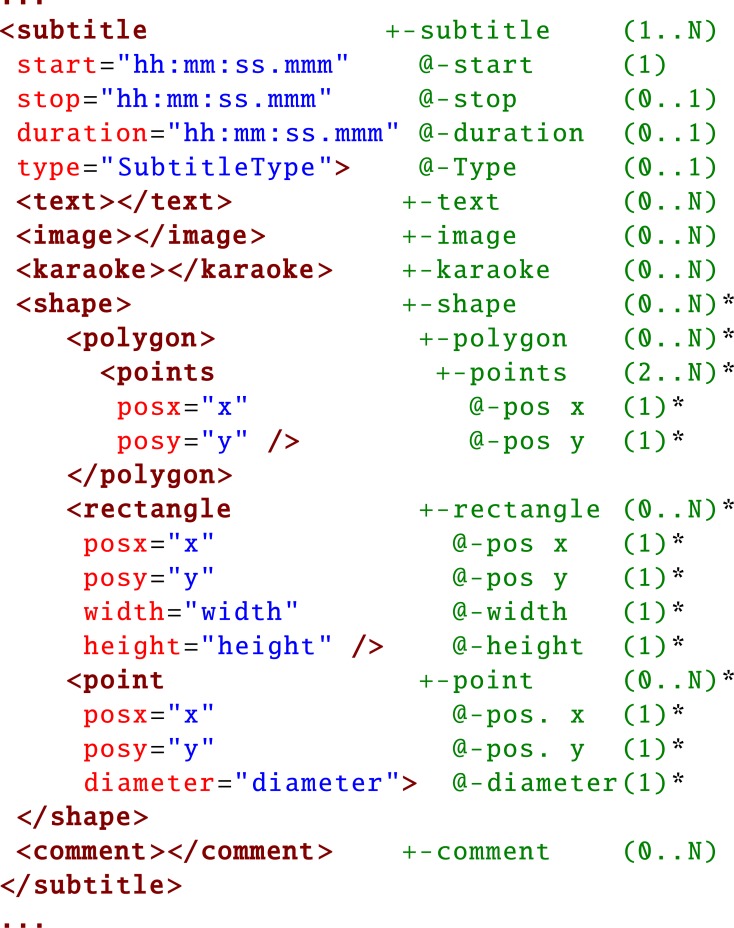
Section of the USF specification (15), * marked attributes are added to the specification and implemented in our altered VLC player.

**Figure 2.**
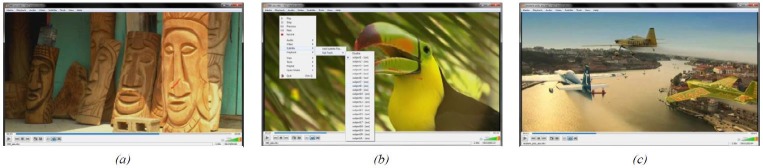
Instantaneous visualization of gaze data, here shown as red dots: (a) visualization of gaze data on a certain frame with VLC player—(b) like normal subtitles one can easily change between the gaze data of subjects—(c) exemplary gaze data on another video sequence.

The so-called subsusf codec module of the VLC player, handles the visualization of all USF content. In detail, this codec module receives streams of the subtitle data for the current frame from the demuxer of VLC and renders a frame overlay at runtime. We extended this module with additional parsing capabilities for our specified shape data, which is then drawn into the so-called subpictures and passed on to the actual renderer of VLC. Since the thread will be called for every frame, the implementation is time-critical, and we decided to use the fast rasterization algorithms of Bresenham ( 5 ). Additionally, we added an option to fill the shapes, which is implemented with the scan line algorithm ( 28 ). In order to ensure that our enhancements of the subsusf module will be available within the next VLC, we submitted our changes to the official VLC 3.0.0 developer repository and suggested an ongoing development of the USF. In Figures 2, 7, and 9, instantaneous visualization of gaze data by the VLC player can be seen.

For testing all features of our implementation, we created a MKV container, containing a dummy video file, as well as USF files with all supported attributes. VLC can open these containers and yields the desired visualization of geometric object annotations ( 20 ). This proves our concept that the incorporation of metadata into USF is possible and that streaming, using MKV as a container format, is also possible, since both content and metadata are integrated as temporal payload. Opening the container in a released version of VLC (2.2.4) without the additional adjustments in the subsusf module will not conflict with the normal video playback, but will not visualize the incorporated annotations, either.

**Metadata as ASS.** Since the USF-based prototype requires a modified version of the VLC media player, a broad audience is still excluded from watching the gaze data set without that player. Therefore, we provide a second prototype based on ASS, a subtitle format with drawing commands, that is widely supported thanks to the free ASS library, and which is also supported by many standalone DVD players. In contrast to USF, the ASS subtitle format cannot carry all of the desired metadata, as it is not capable of representing complex data structures. Due to its design, it is only capable of embedding content that can be visualized, thus, any other content will be lost by the conversion from, e.g., gaze data to ASS.

For generating the lossy ASS files from lossless USF files, a simple translation stylesheet using XSLT (Extensible Stylesheet Language Transformations) has been written. After the conversion, a MKV container is created including the video and one ASS track for each subject. The resulting container makes metadata accessible for a broad audience, as the ASS visualization can be displayed by many video players without the need of modification.

### Muxing Multimedia Containers

At the early stage of this project, we developed an open source command line tool that converts CSV eye tracking and gaze data files to USF files of several subjects, which were then converted to ASS and encapsulated together with the original video stimuli in a single MKV file. Using this scriptable command line tool, we have already converted 319 gaze tracking data sets ( 17, 11, 6, 1 ) for testing, as well as for highlighting the potential of our approach^1^.

**Figure 3.**
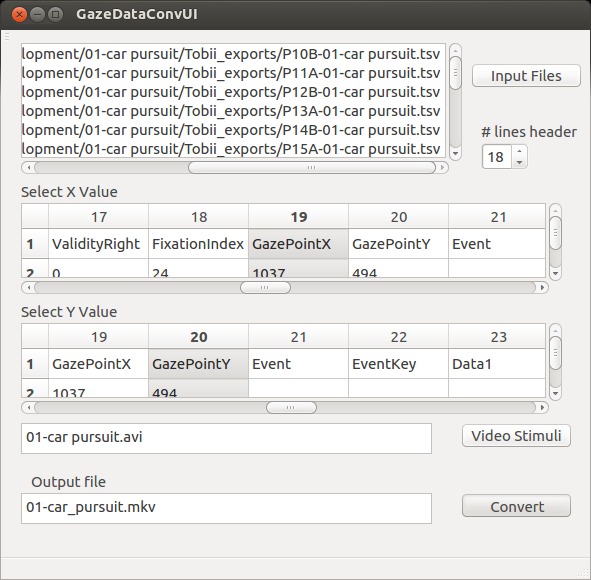
Demuxing a MKV file, an option for experts to extract the raw data for research. In the track select, one can see the tracks for instantaneous visualization (video and subtitle tracks), sonification (audio tracks), and the attachment (general metadata) carrying the raw data.

Unfortunately, the diversity of available gaze data formats requires a huge number of options which must be set for running this command line tool, so that adapting it to another gaze data format takes at least one hour. Therefore, we started to develop a graphical tool for converting gaze data to USF and ASS. Therefrom, these converted files and the stimuli will be mux into MKV containers. As seen in Figure 3, the user interface (UI) implemented in QT is divided into four functional blocks: input file selection, the selection of rows one wants to visualize, the video (stimulus) selection, and the output file naming dialog.

After pressing the button “Input Files”, a file dialog appears in which the user can select one or several gaze files in the same format. All selected files will be listed next to the button. In the next step, the user has to indicate how many lines of the header are in front of the gaze data. If the correct number of header lines is selected, the UI displays the data in two tables—one table to specify the values of the X and one of the Y coordinate. These X and Y coordinates are the center of the circle, which visualizes the gaze points on top the video stimulus. Next, the user defines the stimulus video. After defining an output location, the “Conversion” button becomes active, and the user can start the conversion. After a successful conversion, two MKV containers per gaze data set were created, one based on USF and one based on ASS.

Currently, this tool only supports CSV files and is not able to synchronize gaze data to certain frames. This issue is caused by various time-stamp formats, which differ depending on the eye-tracking hardware used. A further drawback of our first graphical UI is that additional features, like the different visualization of saccades and fixations as seen as in Figure 9(b), are not yet implemented.

### Demuxing Multimedia Containers

**Figure 4.**
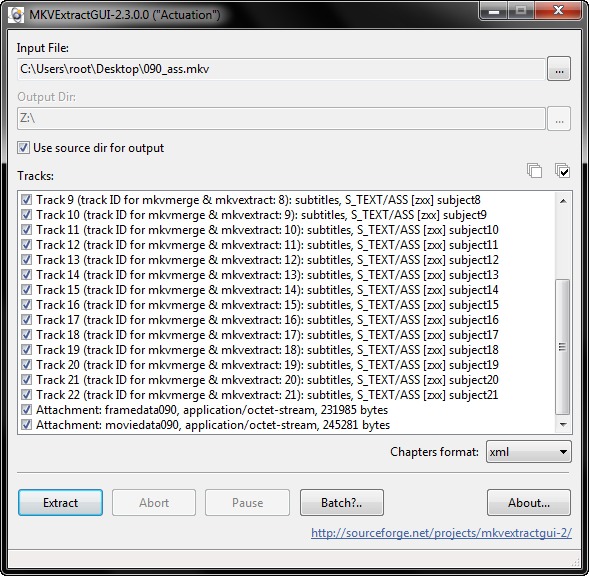
Prototype of the graphical UI for converting gaze data set into MKV container which provides instantaneous visualization.

For expert use, extraction of all data in the MKV container is necessary. For this purpose, the off-the-shelf tool mkvextract ( 13 ) can be used. As illustrated in Figure 4, this standard tool for MKV container handling comes with a graphical UI and a command line UI. Both were developed and are maintained by the MKV community.

### VA Using Standard Multimedia Players

The use of gaze data presented in multimedia containers is quite intuitive, as the user interface builds on metaphors known from entertainment content. Hence, the user can change the visualizations like subtitles. Different types of auditory display can be selected, e.g. audio languages ( 21 ), shown in Figure 2(b). The nature of the general subtitle system in VLC only allows for one active subtitle track at all times, which unfortunately limits the range of possibilities for VA tasks significantly. Since it is often important to visualize gaze-object relations, e.g., to compare the reactions of subjects to a given stimulus, we developed two VLC add-ons for the simultaneous and synchronized playing of different subtitle tracks.

Since such a feature is rarely needed in general contexts and is therefore missing, the VLC player also does not have the required abilities. To improve its usability as a scientific tool, we extend it with this operation commonly needed in the domain of visual analytics. At this point, two VA solutions for providing additional functionalities are discussed.

On the one hand, one might directly extend the source code of the software chosen for the extension if the proposed USF subtitle format is used. Even if the required changes are quite complex and low-level, they will pay off in high performance and in a broad range of dependencies between inner software parts of the application. However, the increase in complexity with the resulting consequences for performance and security is hardly acceptable if just a small fraction of users will need it. Moreover, such a custom patch would require custom compilation of the software until it is sufficiently tested and has found its way into the binary releases of the player. The procedure of translating VLC’s human-readable code into binary executables and linking it to the more than 50 required dependencies on the machine of the user requires advanced knowledge of the system, and its infrastructure, let alone that compilation may require root rights.

On the other hand, one might use some runtime processing to avoid these drawbacks. By default, the VLC multimedia player supports custom add-ons, which are executed on demand and do not need any code compilation. Therefore, it would be possible to have pieces of code that are easy to install, usable, and removable without any problems or residues such as applications on current smartphones. Technically, these add-ons are small text files with the extension LUA stored in scopedependent directories. These scripts contain code written in a slightly outdated version of the cross-platform scripting language LUA, which was designed to provide a fast interface to extend and customize complex software projects like video games after their release. Therefore, LUA extensions match our needs perfectly. We think that the advantages in usage outweigh the in comparison to binary code poorer performance of scripting solutions. Therefore, we built two analysis tools using the dynamic approach. Even if both add-ons may be able to fulfill the same goals of interactive visualization and analysis of different data set items, like the object of interest annotation and the gaze points of participant P1A, at the same time, they still differ. However, not only do they differ in their mode of presentation—one stimulus window versus several stimulus windows—, but also in their advantages and disadvantages of their graphical representation depending on the context of the VA task and the content of the data set.

**Figure 5.**
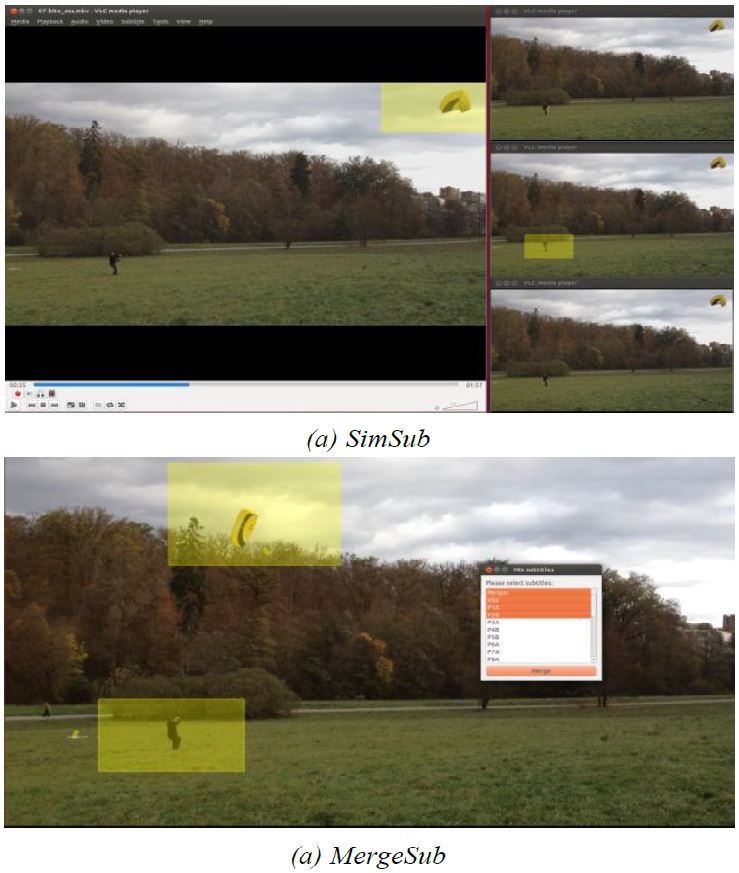
Different areas of applications for the extensions:(a)visualization of gaze data in multiple windows for finding correlations between subjects—(a)visualization of multiple gaze data within a window for detailed analysis.

Our first extension (SimSub), shown in Figure 5(a), allows visualizing different eye-tracking datasets in multiple windows. It may be used to provide a rough overview of the experimental results of multiple participants. A simple click on the requested subtitle tracks creates another instance which behaves exactly like the previous one. The parallel video streams are synchronized within a certain hierarchy. The VLC player started by the researcher works as a controller: Changes in its playback are transmitted in real-time towards the other windows, guaranteeing an exact comparison on a frame basis. The other instances have neither control elements nor timelines, but are purely depending on the “master” window. Technically, the extension uses the fact that the VLC multimedia player provides an interface for remote access. If an eye-tracking dataset should be visualized in parallel, it starts another instance in the background, sets the proper subtitle track, and synchronizes it with the main window. Any modification of the current state, e.g. pausing or stopping is immediately sent via interprocess communication towards all the other instances. This approach allows using all the embedded features of the VLC player regarding its capabilities of many supported subtitle formats. Moreover, the isolated instances may not influence each other in exceptional circumstances such as parsing errors and allow a straightforward VA workflow by a fully customizable order of the visualizations, with the ability to show and hide them on demand. However, the multiple instances may have a performance impact if used in an excessive manner. This restriction arises due to the limitation of the provided LUA interface that makes it necessary to handle some commands in a very cumbersome manner. Hence, our current implementation sometimes lacks correct synchronization of the video and the subtitle between all open views, especially when working with three or more views.

Our second extension (MergeSub) allows visualizing eye-tracking data from different participants using a single instance of the VLC player, which also avoids the synchronization problems (cf. Figure 5(b)). It is designed to find tiny differences in the experimental results of multiple participants, which might become obvious only if played directly side by side. The researcher selects an arbitrary number of subtitle tracks, which are then displayed together on the video sequence. Technically, this ability is provided by an automatic merge of the selected subtitle tracks into a single track, saved in a temporary file (we do not influence the renderer directly). As it is imported afterwards, just like any other subtitle, the researcher has full access to all capacities and advanced functionalities of the VLC player. However, this approach limits the usable subtitle formats dramatically, because it requires a custom merging routine for every format. Our extension so far covers just the formats which are sophisticated enough to visualize eye-tracking data. As a further improvement for this add-on, we plan to assign different colors to the subtitle tracks, which will drastically improve the usability.

With a combination of both techniques, a simple workflow for a basic VA task including reasoning is possible. The dynamic extensibility of the VLC multimedia player would allow further developments regarding the needs of researchers. Thus, an out-of-the-box multimedia player will become a powerful VA software. With its ability for easily available and usable custom extensions, today’s multimedia players are suitable for a variety of VA add-ons, which can be used in parallel or one by one.

## Benefit of Gaze Data in Multimedia Containers

Storing gaze data or other scientific data in multimedia containers will make them accessible to two major groups—the general audience, as well as professionals. Empowered by our approach, almost everyone can visualize and sonificate multimodal metadata. This makes fields like gaze movement research more accessible, understandable and exciting. Thus, it will become easier to acquire non-student participants for experiments, and it will reduce the reluctance against expensive test series. For researchers, interdisciplinary projects will become less complex and more ideas of cooperation can be created if the widespread use of multimedia containers for storing metadata becomes common practice. For example, instead of downloading GBs of zipped files and compiling some software only to be able to get an impression what data is available, it is simple to explore the data set online. This type of visualization may even be included in scientific and popular science publications to demonstrate unprocessed data. Moreover, using a common metadata standard will terminate the tedious process of researchers having to adjust their software for every different gaze data set. Finally, if there is a significant amount of data from the same task in the same format available, machine learning techniques like deep learning can be applied on, e.g., subject gaze trajectories to learn a human-like object tracking behavior. Additionally, consumers may profit from more developed features such as highlighting objects everyone was looking at, and hearing-impaired persons may obtain a computer-generated and understandable auditory description of the scene based on gazes.

In order to emphasize these benefits, we will now summarize a first user study, followed by a short overview of already converted data sets and some ideas how auditory display of metadata might extend visualized data.

### User Study

Initial findings indicate that the proposed multimedia containers are suitable for explorative multimodal data analyses with standard multimedia players. Thus, experts, as well as non-experts, can now easily access the data ( 21 ). Both user groups underline that the beauty of our approach is, to get a first expression of the whole data set with standard software. In their opinion, the proposed format serves as a helpful means of communication, so that, e.g., the exploratory investigation of human sensorimotor interaction in natural environments ( 7, 8 ) will be improved.

By extending these initial user interviews, we performed a software user study with nine subjects. The focus of this study is to i) reevaluate the initial findings gained by the interviews ( 21 ), ii) evaluate our first two custom add-ons as to whether and in what manner will they be used for performing a task and iii) collect feedback on which features are missing, which use cases are not considered, and criticisms affecting the usages.

**Methodology.** We designed the user study questionnaire in four parts. The first part served for grouping the users regarding their working and research experiences. Before starting on the second part of the questionnaire, a five-minute introduction and demonstration of our approach was given. We demonstrated the applicability of downloading, instantaneous visualizing of gaze data, and demuxing the multimedia container for getting the stimulus video, audio as well as the raw gaze data, on the fourth video sequence of Coutrot and Guyader ( 6 ) showing a moving bell. In addition to gaze data and to make the participants think outside the box, the sonification of EEG data ( 21 ) was also illustrated. Based on this introduction, the participants rated on a Likert scale, in the questionnaires’ second section, how different target groups, here unskilled users, domain experts and experts from other domains, might benefit from providing metadata in multimedia containers. The last question of this section asked the participants to brainstorm and collect possible use cases of the demonstrated approach.

The third part of the questionnaire was focused on our two VLC add-ons for the exploitative gaze data analysis. Similar to the previous part, the add-ons SimSub and MergeSub were demonstrated first. The visualization of multiple object annotations in multiple windows (cf. Figure 5(a)) was explained on the air plane video sequence ( 20 ) by showing the annotations of the three airplanes, as well as the river—each annotation in a single playback window. We then introduced the second addon, the merge of multiple gaze data on top of the stimulus video (cf. Figure 5(b)), using the data set ball game ( 11 ). Afterwards, the participants performed three tasks. In the first task, the participants evaluated whether the data set car pursuit ( 11 ) suffers from measurement errors based **on the MKV encoded version.** In the second task, the participants reevaluated their statement of task one **on the original** data set, i.e., on the raw stimulus video file, the annotation file, as well as the eye-tracking files from the 25 observers. For this task, the participants were allowed only the same amount of time as they needed for the first task. In the third task, the participants made statements about the general visual attention behavior, as well as the most focused object on the second video of Riche et al. ( 17 ), which is encoded by our proposed format (cf. Figure 7).

The last part of the user study presented four closing questions. The first two questions queried the data accessibility of our approach, including our add-ons against the data accessibility of the original, non-converted, data set. Then, a question followed on missing or desirable features for improving our approach. Finally, we asked for general statements, criticisms, and ideas.

**Results.** Out of all participants, three had already worked with gaze data and performed research on data analysis related topics. The other six participants never worked with gaze data before and had various kinds of research interests, like deep learning, computer vision or quantum chemistry.

**Figure 6.**
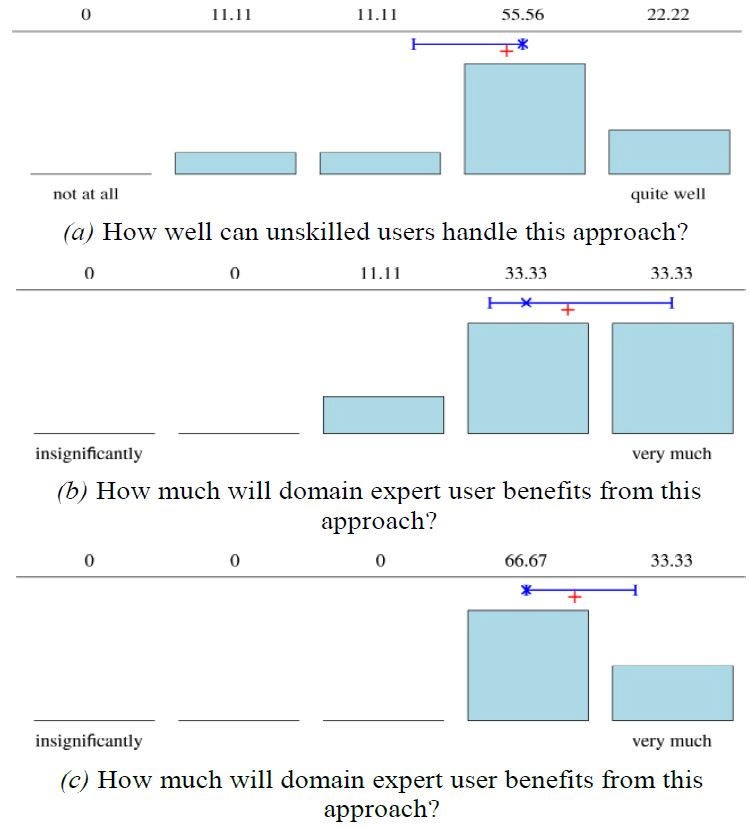
Results based on the opinions of the participants about how different audiences (cf. (a)–(c)) might benefit from our approach, storing metadata in multimedia container. n=9 for (a) and (c), n=7 for (b). Mean marked by +, median by x, quantiles by I.

Figure 6 summarizes the first major assessments of the participants after being introduced to metadata in multimedia containers. All of the participants felt able to indicate the significance of the approach to non-experts and experts from non-sensory data domains. For the experts in the sensory domain, only two participants were not able to indicate their assumptions. Participants suggested many potential use cases: sharing EEG and eyetracking data in a simple and useful way, demonstrating to the general public what the technology does, gaining new insights from the data through novel visualizations, inspecting commercials in customer behavior studies, giving domain experts a feeling for their data, presenting data in a dynamic way, for e.g. talks, get first impression of raw data, and improve e-learning.

**Figure 7.**
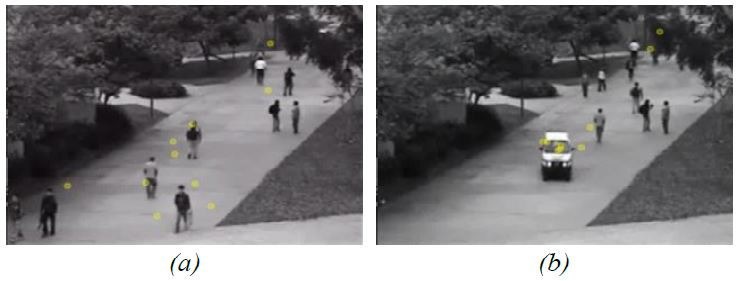
Fourth video sequence of (17) with visualized gaze tracks—yellow dots—from all ten subjects simultaneously by using MergeSub. (a) non-uniform behavior, subjects look at different objects. (b) most subjects focused their view on the car.

Within the practical tasks, seven of nine participants answered the first task completely correct and two partially. On an average, all participants solved the task in two and a half minutes. For example, one participant answered this task as follows: “When the car drives through the roundabout, the calibration of the eye-tracker seems to be off. All subjects look to the similar areas below the car.” By reevaluating their statements on the original unconverted data set, all participants stated that this is impossible without scripts and programming. In the last task, illustrated in Figure 7, eight of nine answered correctly, that in the beginning, the subjects look at different objects (non-uniform behavior) and, as soon as the car entered the scene, nearly all of the participants focused on the car and followed it. Note that for coping with tasks 1 and 3, all participants only used the add-on MergeSub. The add-on SimSub was never used.

**Figure 8.**
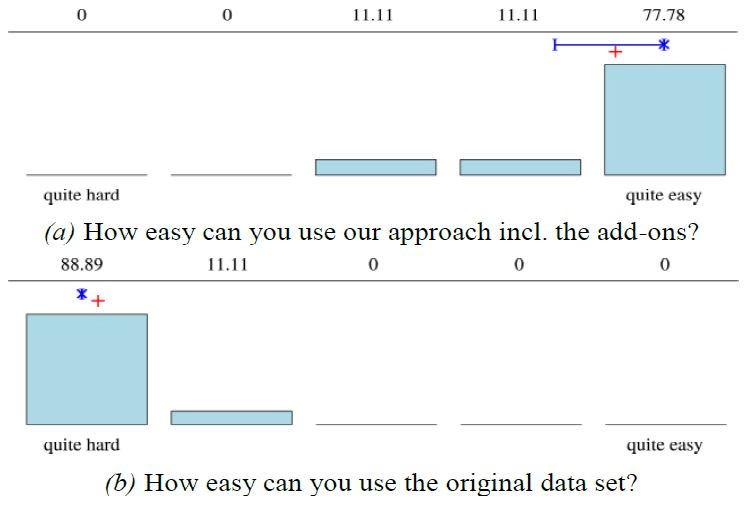
Result of the Likert poll about the usability assessed directly after the practical task. For all sub-figures n=9. Mean marked by +, median by x, quantiles by I.

Within the closing section of the questionnaire, the usability based on the three practical tasks was assessed by all participants. Figure 8 illustrates the results. As possible improvements for the MergeSub add-on, four participants recommended assigning different colors to each metadata track. In their opinion, the visualization would then yield even more useful insights. In addition, to that improvement, one participant suggested the user could click on, e.g., a gaze point or an object annotation to quickly extract quantitative information such as coordinates and time. As criticism, the participants listed the fact that the VLC Player resets the selected subtitle at the end of the playback. Thus, one had to recreate the setting used for the analytics task again and again. Further, one participant argued that the current add-ons are yet not stable so that sometimes rendering errors occur. Finally, the participants remarked that it is a “very interesting and nice product” as well as it is “very useful for researchers in eye-tracking and human behavior studies.”

### Available Data Sets

We have already converted the first 319 video sequences, including their metadata, into MKV containers for promoting their use. The metadata of 319 data sets are available in both the USF and the ASS format. Thus, working with the USF version, there is still access to all data, while the ASS version visualized instantaneously with almost every currently available video player.

**Figure 9.**
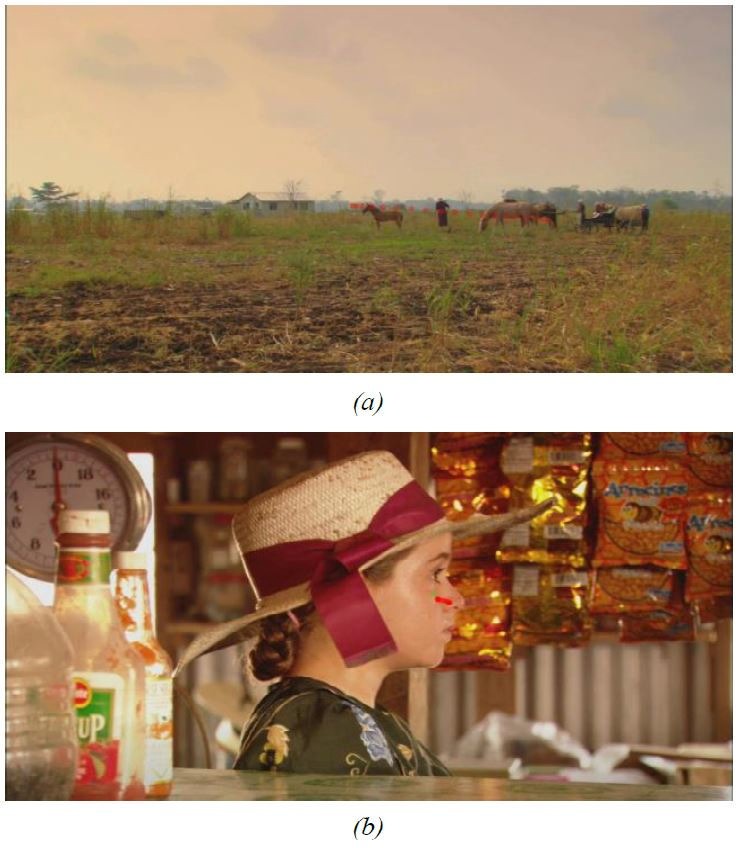
Visualized gaze data by a reused subtitle track on a video by a standard multimedia player. The gaze position on the frames is visualized by a red circle with its diameter depending on the relative pupil size. Green squares visualize fixations. In this example, the sampling rate of the eye-tracker is higher than the frame rate of the video. Thus, multiple points and squares are shown.

Currently, five converted data sets with visualizations can be downloaded^1^. The first converted data set ( 1 ), seen in Figure 5, contains eleven video stimuli together with gaze trajectories of multiple subjects and annotated objects. The second data set ( 6 ) contains 60 video stimuli including gaze data of 18 participants influenced by four auditory conditions. The third data set ( 17 ) shows four different classes of content within the video stimuli, while gazes of ten subjects were tracked (cf. Figure 7). To evaluate the scalability of our approach, the 214 stimuli and the corresponding gaze trajectories of a data set ( 1 ) were converted, showing a short video sequence and a freeze frame from it (example frames are shown in Figures 2(a), 2(b), and 9). Further, as an example from outside the gaze tracking domain, we converted seven sequences of the Berkeley Video Segmentation Dataset (BVSD) ( 23 ) proving bounding-boxes, as well as pixelaccurate annotation of several objects, illustrated in Figure 2(c).

### Auditory Display of Metadata

The sixth available dataset provides, in addition to visualization of gaze points, an auditory display of the alpha rhythm from the raw EEG data of a participant. For transferring the resulting alpha power into sound, two different approaches were taken. For the frequency modulation, a carrier frequency of 220Hz—corresponding to note a—was modulated in its frequency by the power of the alpha signal. For the volume modulation, the same carrier frequency was modulated in its power with respect to the alpha power, with a louder tone meaning a stronger power. As visualized in Figure 1, the audio tracks are reused for muxing the resulting audio streams as WAV files into the MKV container.

## Conclusion

The importance of gaze data in general, and especially the amount of VA applications, will significantly increase, once gaze data, as well as other metadata, are provided in multimedia containers. We have demonstrated instantaneous visualization using these multimedia containers with common video players. By extending today’s multimedia player with add-ons, such as our addons for multi-subtitle visualization, simplistic VA is also possible. Hence, no specific VA software is needed and no new interaction metaphors must be trained. Familiar metaphors are used in our add-ons, e.g. metaphors like switching subtitles. In the long term, and as a result of the data format comparison in Table 1, the research community and the industry should seek to establish a standard based on MPEG-7 for recording, exchanging, archiving, streaming and visualizing, gaze as well as other metadata. Due to its proper specification, MPEG-7 provides a platform for consistent implementations in all media players, but due to its specification, the implementation and testing will require many resources. By muxing MPEG-7 into MP4 containers, streaming, visualization, and auditory display are guaranteed. Unfortunately, we recognize a lack of MPEG-7 standard libraries and integration in current media players. In order to quickly close this gap, we have presented ad-hoc prototypes allowing us to promote embedding metadata into multimedia containers, leading to the immediate use of the metadata. Our approach reuses the USF subtitle format to encode eye-tracking data, allowing to visualize them in a patched version of the popular VLC media player. For other media players, as well as for standalone DVD players, we provide the ASSbased multimedia container. To motivate the VA software designers also to develop VA add-ons based on existing software, we provide two VLC player add-ons, which allow the reasoning if, e.g., two gaze trajectories of two different subjects are focused on the same object of interest. For the future, we plan to expand VA add-ons to support almost every kind of scientific metadata and to use the insights gained for bio-inspired, as well as, sensory-improved computer vision ( 19 ).

**Table 1:** Comparison of the USF-based prototype, the ASS-based prototype, and the MPEG-7 approach. 1 indicate full, ½ indicate partially, and 0 indicate non-support.

	USF prototype	ASS prototype	MPEG-7prototype
General metadata representation	1	½	1
Supporting complex metadata	½	0	1
Out of the box media player integration	½	1	0
Parallel visualization of different metadata entries	0	0	½
Easy accessibility of metadata with scientific tools	1	½	1
Open source development tools available	1	1	½
Encapsulation in a single file container	1	1	1
Streamable container with temporal data	1	1	1
Formats supported for encapsulation	MKV	MKV, MP4, OGG,...	MP4

## Acknowledgements

We acknowledge support by Deutsche Forschungsgemeinschaft (DFG) and Open Access Publishing Fund of Osnabrück University.

This work was funded by Deutsche Forschungsgemeinschaft (DFG) as part of the Priority Program “Scalable Visual Analytics” (SPP 1335).

## Footnotes

Demonstration video, software including all tools, and converted eye-tracking data sets can be downloaded at https://ikw.uos.de/%7Ecv/projects/mm-mkv
